# Angiogenic T cells and cognitive function in older adults with type 2 diabetes treated with GLP-1 receptor agonists

**DOI:** 10.1007/s40520-026-03417-0

**Published:** 2026-05-24

**Authors:** Miriam Longo, Paola Caruso, Maria Chiara Auriemma, Antonietta Maio, Irene Di Meo, Lorenzo Scappaticcio, Maria Ida Maiorino, Giuseppe Bellastella, Maria Rosaria Rizzo, Giuseppe Paolisso, Katherine Esposito

**Affiliations:** 1 Department of Life Science, Health, and Health Professions, Link Campus University, Rome, Italy; 2 Department of Advanced Medical and Surgical Sciences, University of Campania “Luigi Vanvitelli”, Naples, Italy; 3 Division of Endocrinology and Metabolic Diseases, AOU University of Campania “Luigi Vanvitelli”, Naples, Italy; 4 Division of Geriatrics and Internal Medicine, AOU University of Campania : “Luigi Vanvitelli”, Naples, Italy

**Keywords:** Type 2 diabetes, Cognitive function, Angiogenic T cells, GLP-1RA, Endothelial dysfunction

## Abstract

**Background:**

Older adults with type 2 diabetes mellitus (T2DM) are at high risk of both cardiovascular complications and cognitive decline, with major implications for independence and self-management. Endothelial dysfunction and impaired angiogenic capacity may play a key role. This study investigated the association between circulating angiogenic T cells (Tang cells) and cognitive function in older adults with T2DM and explored the potential impact of glucagon-like peptide-1 receptor agonist (GLP-1RA) therapy.

**Methods:**

A cross-sectional study was conducted on 154 T2DM patients aged 60–80 years, treated either with GLP-1 receptor agonist (GLP-1RA) plus metformin or metformin alone. Cognitive function was assessed using the Mini-Mental State Examination (MMSE) and Montreal Cognitive Assessment (MoCA). Circulating CD3+CD31+CXCR4+ Tang cells were quantified by flow cytometry. Propensity score matching was applied to control for age, body weight and HbA1c.

**Results:**

In the overall cohort, higher Tang cell levels were significantly associated with better cognitive performance (MoCA, r = 0.423; MMSE, r = 0.428; both P < 0.001). After matching, 35 patients in each treatment group were included in the comparative analysis. The GLP-1RA + MET group showed significantly higher circulating Tang cell levels than the MET group, both in absolute counts and as percentage of CD3+ T cells (P < 0.001).

**Conclusions:**

Circulating Tang cell levels are positively associated with cognitive function in older adults with T2DM. GLP-1RA therapy is associated with higher Tang cell levels compared with metformin alone, suggesting a potential association with mechanisms related to endothelial repair in diabetes-related cognitive impairment in older age.

**Supplementary Information:**

The online version contains supplementary material available at 10.1007/s40520-026-03417-0.

## Introduction

Type 2 diabetes mellitus (T2DM) is a complex chronic metabolic disorder highly prevalent in older adults. It is associated with an increased burden of cardiovascular and microvascular complications, disability and loss of independence [1, 2]. In this age group, the coexistence of multimorbidity, polypharmacy and functional impairment makes diabetes management particularly challenging [3]. Beyond its vascular impact, T2DM is increasingly recognized as a major risk factor for cognitive decline and dementia, especially in older individuals [4]. Cognitive impairment in people with T2DM has important clinical consequences, as it may compromise treatment adherence, increase the risk of hypoglycemia and accelerate functional dependence [5]. However, the precise mechanisms linking T2DM to cognitive decline remain incompletely understood, with chronic low-grade inflammation, endothelial dysfunction and neurovascular unit impairment emerging as central contributors [6].

Endothelial dysfunction represents an early step in the development of atherosclerosis, and it is implicated in the pathogenesis of both macro- and micro-vascular complications of T2DM [7]. Adequate vascular repair mechanisms are essential to counteract endothelial injury and preserve tissue perfusion, particularly in the ageing vasculature [8]. Circulating angiogenic cells, which include both endothelial progenitor cells (EPCs) and angiogenic T cells (Tang cells), play key roles in repairing damaged endothelium and promoting angiogenesis and neovascularization [9]. Reduced angiogenic activity and aberrant angiogenesis have been associated with cerebral small vessel disease, white matter hyperintensities and cognitive impairment [10].

In recent years, GLP-1 receptor agonists (GLP-1RAs) have emerged as an effective therapeutic option for managing T2DM in older adults, providing not only effective glycemic control but also significant cardiovascular and renal protective effects [11, 12]. These agents have also been suggested to exert neuroprotective actions, possibly through anti-inflammatory effects, improved endothelial function and modulation of neurovascular pathways [13]. In a previous study, older adults with T2DM treated with GLP-1RA in combination with metformin exhibited significantly higher circulating EPCs levels and better cognitive performance compared with those receiving metformin alone [14].

Tang cells, a subset of T lymphocytes characterized by the expression of CD3, CD31, and CD184, play a critical role in vascular repair by supporting EPC differentiation, facilitating the repair of damaged endothelium and stimulating neovascularization [15]. Reduced Tang cell levels have been reported in several conditions associated with endothelial dysfunction, including rheumatoid arthritis [16], hypertension-related cerebral small vessel disease [17] and T2DM [18]. While the association of EPCs with cognitive function has been established, the relationship between Tang cells and cognitive function in T2DM remains underexplored. Given the critical role of Tang cells in endothelial function, which seems to be increasingly linked to cognitive performance, this study aims to investigate the association between circulating levels of Tang cells and cognitive outcomes in individuals with T2DM. A secondary objective was to explore whether treatment with GLP-1RA plus metformin is associated with a more favorable angiogenic T-cell profile compared with metformin alone in this population.

## Methods

This cross-sectional study was conducted at the University of Campania "Luigi Vanvitelli" (Naples, Italy) and included community-dwelling older adults with T2DM. The study design and main inclusion criteria have been previously described [14]. Briefly, patients aged between 60 and 80 years, with type 2 diabetes duration > 5 years, with HbA1c ≥ 7% and < 8.5%, and in treatment for at least 12 months with GLP-1RA plus metformin (GLP-1RA + MET group) or metformin alone (MET group) according to clinical practice were included in the analysis. Exclusion criteria comprised acute illness, recent cardiovascular events, severe psychiatric disease, known dementia, major neurological disorders, unstable medical conditions and any condition potentially interfering with cognitive testing or blood sampling. The study protocol was approved by local ethical committee (n. 625/2018 of 12.09.2018) and conducted according to the principles of the Helsinki Declaration II. All participants provided written informed consent before enrolment.

All participants underwent a detailed clinical evaluation, including medical history, physical examination and assessment of cardiovascular risk factors and complications. Clinical and metabolic variables, including age, sex, body mass index (BMI), blood pressure, diabetes duration, history of cardiovascular events and current medications, were recorded. Fasting blood samples were used to determine HbA1c and other routine biochemical parameters according to standard laboratory methods.

### Cognitive assessment

Cognitive function was assessed using a comprehensive geriatric cognitive evaluation. Global cognition was measured by the Mini-Mental State Examination (MMSE), corrected for age and educational level, and by the Montreal Cognitive Assessment (MoCA), which explores multiple cognitive domains including memory, executive function, attention and visuospatial abilities. Tests were administered by trained personnel in a quiet setting, following standardized procedures. Higher scores indicate better cognitive performance for both MMSE and MoCA.

### Assessment of tang cells

Circulating Tang cells were measured by flow cytometry. Peripheral blood mononuclear cells (PBMCs) were isolated from venous blood samples by density-gradient centrifugation using Ficoll. Cells were incubated at 4°C for 30 min with fluorochrome-conjugated monoclonal antibodies, including anti-CD3-FITC, anti-CD31-PE and anti-CD184-Cy5 (CXCR4). Data acquisition was performed on a FACSCalibur flow cytometer (Becton–Dickinson), with 500,000 events recorded per sample.​ Briefly, lymphocytes were first identified based on forward and side scatter properties, followed by doublet exclusion. CD3⁺ T cells were then selected, and angiogenic T cells were defined according to the expression of [CD3^+^CD31^+^CD184^+^] within the CD3⁺ population. The gating strategy was applied consistently across all samples using a standardized protocol.

In addition, intra-assay variability was assessed by repeated measurements of the same sample within the same analytical run (triplicate acquisitions). The coefficient of variation (CV), calculated as the standard deviation divided by the mean and expressed as a percentage, was approximately 10–11%. This level of variability is considered acceptable for flow cytometric analysis of rare cell populations. Within this subset, CD3^+^CD31^+^CD184^+^ Tang cells were quantified and expressed both as absolute counts (cells/ml) and as percentage of total CD3^+^ T cells.

### Statistical analysis

Continuous variables are presented as mean ± standard deviation or median (interquartile range) according to their distribution, and categorical variables as counts and percentages. Correlations between Tang cell levels and cognitive scores (MoCA and MMSE) in the overall cohort were evaluated using Pearson or Spearman correlation coefficients, as appropriate.​ To compare Tang cell levels between treatment groups while reducing confounding, propensity score matching was performed. The propensity score was calculated for each participant based on age, body weight HbA1c and prior CV events, specifically history of myocardial infarction, stroke, and peripheral vascular disease, and patients treated with GLP-1RA + MET were matched to those receiving MET alone using a caliper width equal to 0.2 of the standard deviation of the logit of the propensity score. The balance of covariates after matching was assessed using the standardized difference method.

Between-group comparisons were carried out using the independent two-sample t-test or Mann–Whitney U test for continuous variables, and the chi-square test for categorical variables, as appropriate. Variables with non-normal distribution were log-transformed before analysis. A multivariable linear regression analysis was performed with MoCA score and MMSE score as the dependent variables and Tang cell counts, age, sex, BMI, HbA1c and diabetes duration as independent variables, to assess whether Tang cells were independently associated with cognitive performance*.* A two-sided P value < 0.05 was considered statistically significant. All analyses were conducted using Stata, version 16.0 (Stata Corp, College Station, TX, USA).

## Results

A total of 154 older adults with T2DM were included, 78 treated with GLP-1RA plus metformin (GLP-1RA + MET group) and 76 with only metformin (MET group). The median age of the overall cohort was 69 years, with a median BMI 30.2 kg/m^2^, and a median HbA1c level 7.2% (55 mmol/mol). Thirty-nine percent of participants were women, and 25% had a previous cardiovascular event. Median MMSE and MoCA scores were 27 and 24, respectively. Baseline clinical and metabolic characteristics are reported in Table 1.

**Table 1:** Clinical and metabolic characteristic of overall participants in the study

	**Overall** **(N = 154)**
Age, years	69 (66, 75)
Duration of diabetes, years Female gender, n (%)	15 (8, 25) 60 (39)
Fasting glucose, mg/dl	114 (101, 141)
HbA1c, %	7.2 (7.0, 7.6)
HbA1c, mmol/mol	55 (53, 60)
Weight, Kg	83.5 (75, 95)
BMI, Kg/m^2^ WC, cm	30.2 (27.7, 33) 100 (90, 105)
SBP, mmHg	130 (120, 140)
DBP, mmHg HR, bpm/min Total Cholesterol, mg/dl HDL-Cholesterol, mg/dl LDL- Cholesterol, mg/dl Triglycerides, mg/dl Creatinine, mg/dl	80 (75, 80) 75 (68, 84) 157 (135, 181) 49 (42, 62) 81 (68, 108) 107 (77, 150) 0.86 (0.7, 1.0)
eGFR, ml/min/1.73m^2^ UACR, mg/g CRP, mg/L Diabetic retinopathy, n (%) Diabetic kidney disease, n (%) Diabetic neuropathy, n (%) Prior cardiovascular event, n (%)	79 (64, 89) 10 (8, 50) 1 (0.6, 1.7) 34 (22) 24 (15) 18 (16) 39 (25)
MoCA	24 (19, 28)
MMSE	27( 24.4,29)

BMI, body mass index; WC, waist circumference; SBP, systolic blood pressure; DBP, diastolic blood pressure; HR, heart rate; HDL, high-density lipoprotein; LDL, low-density lipoprotein; eGFR, estimated glomerular filtration rate; UACR, urinary albumin-to-creatinine ratio; CRP, C-reactive protein; MoCA, Montreal Cognitive Assessment; MMSE, Mini-Mental State Examination; HbA1c, glycated hemoglobin.

**Fig. 1:**
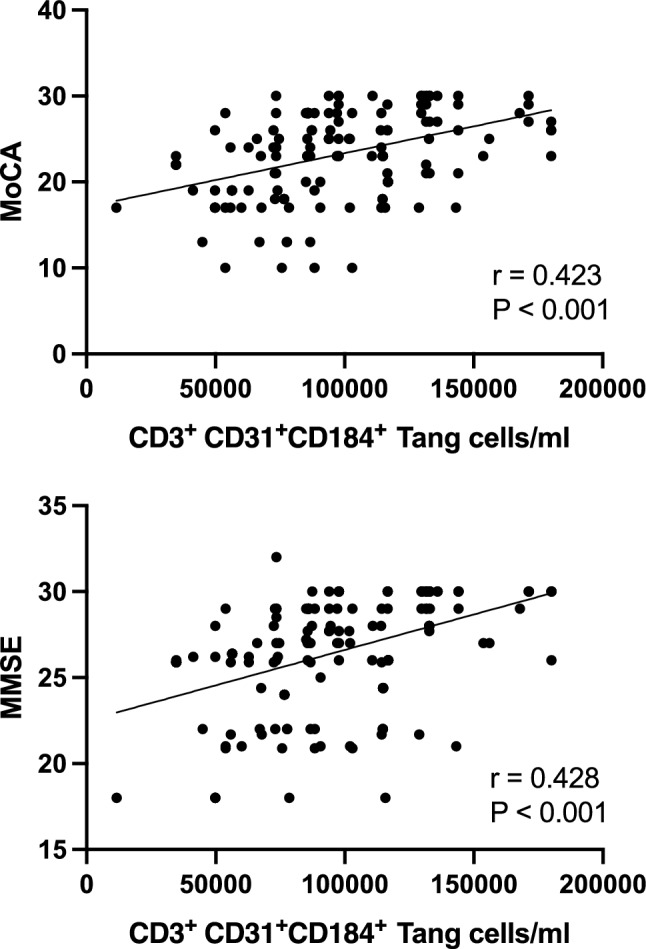
Statistical associations between cognitive test scores and circulating levels of Tang cells by univariate analysis

In the overall cohort, circulating Tang cell levels showed a significant positive correlation with cognitive performances. Higher Tang cells counts were associated with higher MoCA (r = 0.423, p < 0.001) and higher MMSE (r = 0.428, p < 0.001) scores (Fig. 1).

To assess whether this association was independent of major clinical variables, a multivariable linear regression model was fitted with MoCA score and MMSE score as the dependent variables and Tang cell counts, age, sex, BMI, HbA1c, diabetes duration and GLP-1RA therapy as independent. In the model with MoCA as the dependent variable, Tang cell counts remained independently and positively associated with cognitive performance (β = 0.251; 95% CI 0.05 to 0.45; p = 0.016). Age (β =  − 0.210; 95% CI − 0.321 to − 0.099; p < 0.001) and GLP-1RA therapy (β = 3.853; 95% CI 2.368 to 5.338; p < 0.001) were also independently associated with higher MoCA scores Table 2.

**Table 2:** Multivariable linear regression analyses assessing the independent association between MoCA score and Tang cells count, age, sex, BMI, diabetes duration and GLP-1RA therapy

Variable	β coefficient	95% CI	P
Tang cells count (cells/ml)	0.251	0.05 – 0.45	0.016
Age	-0.210	-0.321 – -0.099	< 0.001
Sex	1.950	-0.639 – 3.261	0.784
BMI	-0.036	-0.173 – 0.101	0.607
Diabetes duration	0.113	-0.035 – 0.191	0.075
GLP-1RA therapy	3.853	2.368 – 5.338	< 0.001

In the model with MMSE as the dependent variable, Tang cell counts were independently and positively associated with cognitive performance (β = 0.171; 95% CI 0.04 to 0.31; p = 0.013). Diabetes duration (β = 0.062; 95% CI 0.011 to 0.114; p = 0.019) and GLP-1RA therapy (β = 3.340; 95% CI 2.360 to 4.320; p < 0.001) were also independently associated with MMSE scores.

In contrast, age showed a borderline inverse association (β =  − 0.070; 95% CI − 0.143 to 0.004; p = 0.065), while sex (β = 0.355; 95% CI − 0.509 to 1.219; p = 0.422) and body mass index (β =  − 0.047; 95% CI − 0.137 to 0.043; p = 0.313) were not significantly associated with MMSE scores (Table 3).

**Table 3:** Multivariable linear regression analyses assessing the independent association between MMSE score and Tang cells count, age, sex, BMI diabetes duration, GLP-1RA therapy

Variable	β Coefficient	95% CI	p-value
Tang cells count (cells/ml)	0.171	0.04 – 0.31	0.013
Age	-0.070	-0.143 – 0.004	0.065
Sex	0.355	-0.509 – 1.219	0.422
BMI	-0.047	-0.137 – 0.043	0.313
Diabetes duration	0.062	0.011 – 0.114	0.019
GLP-1RA therapy	3.340	2.360 – 4.320	< 0.001

**Fig. 2:**
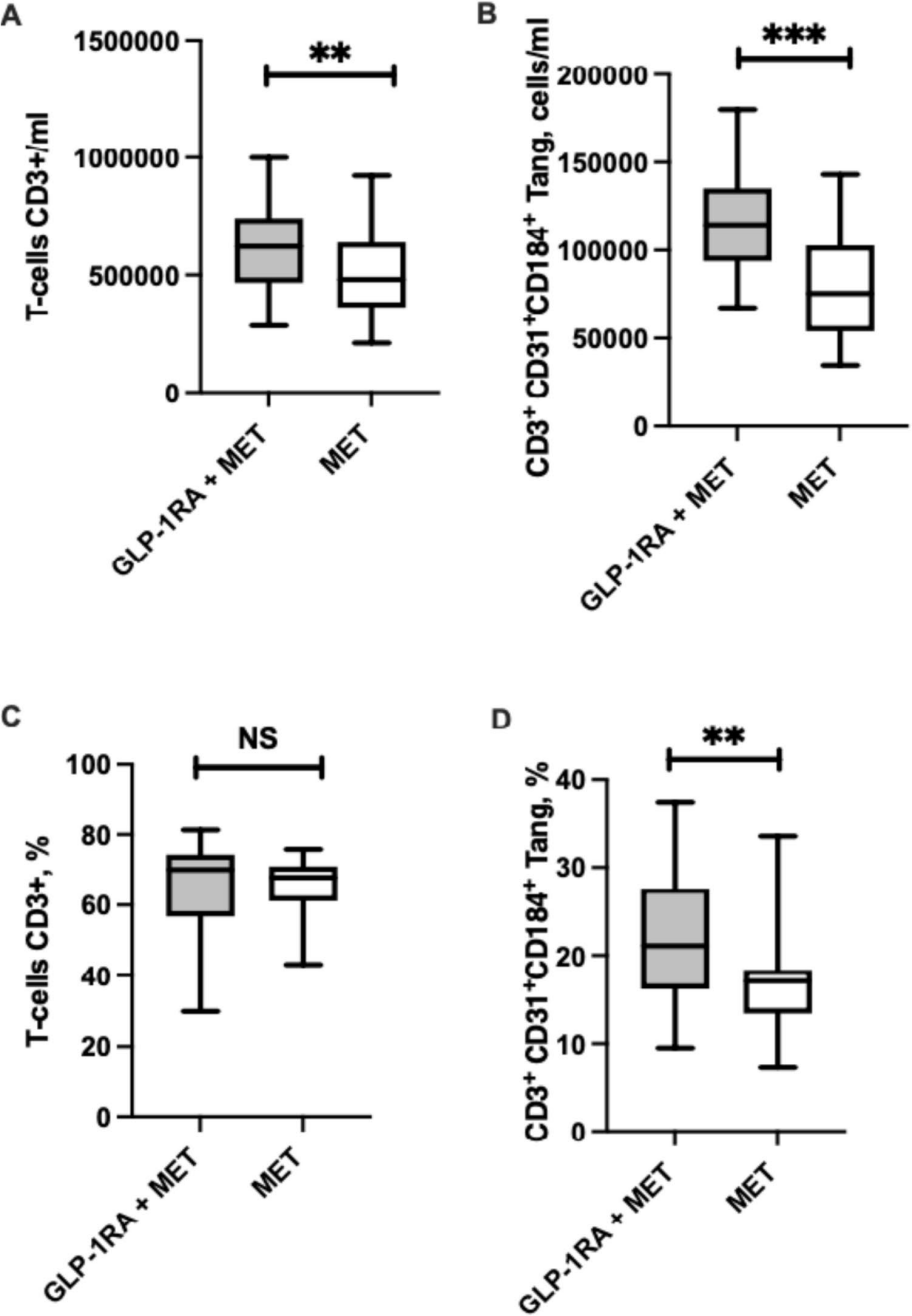
Box plots with median and error bars for counts of T-cells CD3^+^ and angiogenic T-cells. Significant differences are indicated. ** P < 0.05, *** P < 0.001. **A**: Absolute number of CD3^+^ cells in patients of the GLP-1RA + MET Group and of the MET Group; **B**: Absolute number of Tang in patients of the GLP-1RA + MET Group and MET Group; **C**: Percentage of CD3^+^ cells in patients of the GLP-1RA + MET Group and MET Group; **D**: Percentage of Tang in patients of the GLP-1RA + MET Group and MET Group

After matching patients in the two groups for age, weight, HbA1c and prior CV events, we obtained 35 patients in the GLP-1RA + MET group and 35 in the MET Group (Supplementary Table S1). The percentage of Tang, together with MoCA and MMSE scores, were significantly higher in the GLP-1RA + MET group than in the MET group. In particular, Fig. 2 shows the data for CD3^+^ T cells and CD3^+^ CD31^+^CD184^+^ Tang cells levels across the two patient groups. Patients in the GLP-1RA + MET group exhibited significantly higher levels of CD3^+^ T cells, in absolute count but not in percentage, compared to those in the MET group [620,000 (467,000–727,000) vs. 480,000 (363,000–635,000) cells/ml, P = 0.028; and 70% (57–74) vs. 68% (61–71), P = 0.207] (Panel A and C). Similarly, levels of circulating CD3^+^ CD31^+^CD184^+^ Tang cells were markedly more elevated, both in absolute count and in percentage, in the GLP-1RA + MET group [115,000 (94,000–134,000) vs. 76,000 (54,000–102,000) cells/ml, P < 0.001; and 21.1% (16–28.6) vs. 17% (13.4–18.3), P = 0.004] than MET group (Panel B and D).

## Discussion

In this cross-sectional study, circulating Tang cells were positively associated with cognitive performance in older adults with T2DM. Circulating Tang cell counts showed significant correlations with both MoCA and MMSE scores and remained independently and positively associated with these cognitive measures after adjustment for major clinical and metabolic variables, including GLP-1RA therapy. In addition, treatment with GLP-1RAs in combination with metformin was associated with significantly higher Tang cell levels compared with metformin alone. The stronger association observed with MoCA, together with the independent inverse effect of age, is consistent with the higher sensitivity of this tool in detecting early cognitive impairment. These findings suggest that Tang cells may capture a specific aspect of neurovascular integrity that is particularly relevant in the aging diabetic population.

Cognitive impairment in older adults with T2DM is increasingly recognized as the result of complex interactions between metabolic dysregulation, vascular damage, and age-related neurodegenerative processes [19]. Endothelial dysfunction and impaired microvascular repair play a central role in this context. Our results support the hypothesis that angiogenic competence, as reflected by circulating Tang cell levels, is associated with cognitive health in this vulnerable population. Previous studies have shown that GLP-1RA therapy is associated with improved endothelial function, increased circulating endothelial progenitor cells (EPCs), and better cognitive performance in individuals with T2DM [14]. EPCs are bone marrow–derived cells that contribute to vascular repair and maintenance, and their reduction has been associated with increased cardiovascular risk and cognitive impairment in diabetes [20, 21]. In particular, circulating CD34⁺KDR⁺CD133⁺ EPCs have been identified as independent predictors of cognitive function in patients with T2DM [14]. In line with these observations, the present study shows that higher levels of CD3⁺CD31⁺CD184⁺ Tang cells are associated with better cognitive performance, further supporting the link between angiogenic mechanisms and brain health. Tang cells play a key role in vascular homeostasis by supporting EPC function and promoting endothelial repair. Reduced Tang cell levels have been reported in several conditions characterized by endothelial dysfunction, including rheumatoid arthritis [16], hypertension-related cerebral small vessel disease [17], and T2DM [18]. Neuroimaging studies have linked impaired angiogenic activity to markers of cerebral small vessel disease, such as white matter hyperintensities, which are highly prevalent in older adults and are known contributors to cognitive decline [10]. Although cognitive consequences may not always be evident in early stages, these findings suggest that angiogenic dysfunction may be involved in the progression of neurovascular damage over time. In a previous study involving 41 patients with T2DM and matched healthy controls, patients with diabetes exhibited reduced Tang cell levels compared to controls [18]. Treatment with the DPP-4 inhibitor linagliptin over 26 weeks was associated with an increase in circulating Tang cells, suggesting that pharmacological interventions may influence angiogenic immune profiles [18]. In our current study, the percentage of Tang cells observed in the GLP-1RA + MET group [21.1% (16–28.6)] closely mirrors the levels found in healthy controls from this last study [20.4% (16.6–27.3)] [18], supporting the hypothesis that GLP-1RA therapy is associated with a more favorable angiogenic profile. The mechanisms underlying the association between GLP-1RA therapy and increased Tang cell levels remain to be fully clarified. GLP-1RAs exert pleiotropic effects beyond glycemic control, including anti-inflammatory actions and improvements in endothelial function, which may influence the mobilization or survival of angiogenic immune cells [22, 23]. Despite the use of propensity score matching, differences in cognitive scores between treatment groups persisted after matching, with higher MoCA and MMSE scores in the GLP-1RA + MET group. This pattern suggests that residual confounding or unmeasured baseline characteristics may still contribute to the observed between-group differences. Therefore, the superior cognitive performance in the GLP-1RA-treated group should not be interpreted as proof of a direct neurocognitive benefit of GLP-1RAs, but rather as potentially reflecting a more favorable overall clinical or neurovascular profile that is only partially captured by the variables included in the matching procedure. Importantly, in multivariable models that additionally adjusted for GLP-1RA therapy and other clinical covariates, Tang cell counts remained independently associated with both MoCA and MMSE scores. This supports the robustness of the relationship between Tang cells and cognitive performance, while underscoring that the direction and causality of this association cannot be determined from the present data.

Strengths of this study include the use of a comprehensive battery of validated tests for cognitive evaluation and the assessment of CD3 + T cells and Tang cells by flow cytometry. The comparison between two therapeutic regimens commonly used in clinical practice and the application of propensity score matching further increase the clinical relevance of the findings. However, some limitations should be acknowledged. The cross-sectional design represents the main limitation, as it precludes any inference on temporal relationships or causality between Tang cells, GLP-1RA therapy and cognitive outcomes. In addition, although propensity score matching and multivariable adjustment were applied, residual confounding by unmeasured factors cannot be excluded. The sample size, particularly in the matched analysis, is modest and may limit the generalizability of the results. Longitudinal studies are needed to determine the temporal relationship between Tang cells and cognitive decline, as well as the potential impact of therapeutic interventions.

## Conclusion

Overall, our findings indicate that circulating Tang cell levels are positively associated with cognitive performance in older adults with T2DM and that GLP-1RA therapy is associated with higher Tang cell levels compared with metformin alone. These observations support a potential link between angiogenic immune pathways, vascular health and cognition in this population, but they should be interpreted with caution given the observational nature of the study. Prospective longitudinal studies, ideally integrating detailed neuroimaging and mechanistic assessments, are warranted to clarify the temporal dynamics and clinical implications of the relationship between Tang cells, GLP-1RA therapy and cognitive decline in type 2 diabetes.

## Supplementary Information

Below is the link to the electronic supplementary material.

Supplementary file1

## Data Availability

The data that support the findings of this study are not openly available due to reasons of sensitivity and are available from the corresponding author upon reasonable request.

## References

[1] Dal Canto E, Ceriello A, Rydén L et al (2019) Diabetes as a cardiovascular risk factor: an overview of global trends of macro- and microvascular complications. Eur J Prev Cardiol 26:32 10.1177/2047487319878371 31722562

[2] American Diabetes Association Professional Practice Committee (2026) Older adults: standards of care in diabetes—2026. Diabetes Care 49:S277–S296. 10.2337/dc26-S013 41358888 10.2337/dc26-S013 PMC12690186

[3] Longo M, Bellastella G, Maiorino MI et al (2019) Diabetes and aging: from treatment goals to pharmacologic therapy. Front Endocrinol (Lausanne) 10:45. 10.3389/fendo.2019.00045 30833929 10.3389/fendo.2019.00045 PMC6387929

[4] Gudala K, Bansal D, Schifano F et al (2013) Diabetes mellitus and risk of dementia: a meta-analysis of prospective observational studies. J Diabetes Investig 4:640–650. 10.1111/jdi.12087 24843720 10.1111/jdi.12087 PMC4020261

[5] Kan W, Qu M, Wang Y et al (2025) A review of type 2 diabetes mellitus and cognitive impairment. Front Endocrinol (Lausanne) 16:1624472. 10.3389/fendo.2025.1624472 40831951 10.3389/fendo.2025.1624472 PMC12358275

[6] Biessels GJ, Despa F (2018) Cognitive decline and dementia in diabetes mellitus: mechanisms and clinical implications. Nat Rev Endocrinol 14:591–604. 10.1038/s41574-018-0048-7 30022099 10.1038/s41574-018-0048-7 PMC6397437

[7] Vanhoutte PM, Shimokawa H, Feletou M et al (2017) Endothelial dysfunction and vascular disease: a 30th anniversary update. Acta Physiol (Oxf) 219:22–96. 10.1111/apha.12646 26706498 10.1111/apha.12646

[8] Lacolley P, Avril S, Gáll T et al (2025) Aging in the vascular system: lessons from mechanobiology, computational approaches, and oxidative stress. Cardiovasc Res 121:1566–1581. 10.1093/cvr/cvaf137 40810190 10.1093/cvr/cvaf137

[9] Kul A, Ozturk N, Kurt AK et al (2023) Detection of angiogenic T cells and endothelial progenitor cells in Behçet disease and determination of their relationship with disease activity. Life Basel 13:1259 37374042 10.3390/life13061259 PMC10301090

[10] Callahan CM, Apostolova LG, Gao S et al (2020) Novel markers of angiogenesis in the setting of cognitive impairment and dementia. J Alzheimers Dis 75:959–969. 10.3233/JAD-191293 32390626 10.3233/JAD-191293 PMC8351220

[11] Giugliano D, Scappaticcio L, Longo M et al (2021) GLP-1 receptor agonists and cardiorenal outcomes in type 2 diabetes: an updated meta-analysis of eight CVOTs. Cardiovasc Diabetol 20:189 34526024 10.1186/s12933-021-01366-8 PMC8442438

[12] Giugliano D, Longo M, Signoriello S et al (2022) The effect of DPP-4 inhibitors, GLP-1 receptor agonists and SGLT2 inhibitors on cardiorenal outcomes: a network meta-analysis of 23 CVOTs. Cardiovasc Diabetol 21:42 35296336 10.1186/s12933-022-01474-z PMC8925229

[13] Sabbagh MN, Cummings JL, Ballard C et al (2025) Repurposing glucagon-like peptide-1 receptor agonists for the treatment of neurodegenerative disorders. Nat Aging. 10.1038/s43587-025-01029-3 41419667 10.1038/s43587-025-01029-3 PMC13528608

[14] Longo M, Di Meo I, Caruso P et al (2023) Circulating levels of endothelial progenitor cells are associated with better cognitive function in older adults with glucagon-like peptide-1 receptor agonist-treated type 2 diabetes. Diabetes Res Clin Pract 200:110688. 10.1016/j.diabres.2023.110688 37116797 10.1016/j.diabres.2023.110688

[15] Hur J, Yang HM, Yoon CH et al (2007) Identification of a novel role of T cells in postnatal vasculogenesis: characterization of endothelial progenitor cell colonies. Circulation 116:1671–1682. 10.1161/CIRCULATIONAHA.107.694778 17909106 10.1161/CIRCULATIONAHA.107.694778

[16] Rodríguez-Carrio J, Alperi-López M, López P et al (2015) Angiogenic T cells are decreased in rheumatoid arthritis patients. Ann Rheum Dis 74:921–927. 10.1136/annrheumdis-2013-204250 24399233 10.1136/annrheumdis-2013-204250

[17] Rouhl RP, Mertens AE, van Oostenbrugge RJ et al (2012) Angiogenic T-cells and putative endothelial progenitor cells in hypertension-related cerebral small vessel disease. Stroke 43:256–258. 10.1161/STROKEAHA.111.632208 21980212 10.1161/STROKEAHA.111.632208

[18] de Boer SA, Reijrink M, Abdulahad WH et al (2020) Angiogenic T cells are decreased in people with type 2 diabetes mellitus and recruited by the dipeptidyl peptidase-4 inhibitor linagliptin: a subanalysis from a randomized placebo-controlled trial (RELEASE study). Diabetes Obes Metab 22:1220–1225. 10.1111/dom.14024 32166899 10.1111/dom.14024 PMC7317866

[19] Chen X, Huang Y, Xiong X (2025) Mechanisms underlying cognitive impairment and management strategies in type 2 diabetes. Front Endocrinol (Lausanne) 16:1655768. 10.3389/fendo.2025.1655768 41169480 10.3389/fendo.2025.1655768 PMC12568408

[20] Fadini GP, Boscaro E, de Kreutzenberg S et al (2010) Time course and mechanisms of circulating progenitor cell reduction in the natural history of type 2 diabetes. Diabetes Care 33:1097–1102. 10.2337/dc09-1999 20150295 10.2337/dc09-1999 PMC2858183

[21] Maiorino MI, Bellastella G, Petrizzo M et al (2017) Effect of a Mediterranean diet on endothelial progenitor cells and carotid intima-media thickness in type 2 diabetes: follow-up of a randomized trial. Eur J Prev Cardiol 24:399–408. 10.1177/2047487316676133 27798369 10.1177/2047487316676133

[22] Ros-Madrid I, Cano-Mármol R, Ferrer-Gomez M et al (2025) Anti-inflammatory properties of GLP-1 receptor agonists and other ancillary benefits from a pharmacological perspective. Can J Physiol Pharmacol 103:369–377. 10.1139/cjpp-2025-0148 41086442 10.1139/cjpp-2025-0148

[23] Caruso P, Maiorino MI, Longo M et al (2025) Liraglutide improves peripheral perfusion and markers of angiogenesis and inflammation in people with type 2 diabetes and peripheral artery disease: an 18-month follow-up of a randomized clinical trial. Diabetes Obes Metab 27:3891–3900. 10.1111/dom.16419 40276845 10.1111/dom.16419 PMC12146450

