# Supplementary material for: Angiogenic T cells and cognitive function in older adults with type 2 diabetes treated with GLP-1 receptor agonists
Source: Aging Clin Exp Res. 2026 May 24;38(1):165. doi: 10.1007/s40520-026-03417-0 (PMC13415266; doi:10.1007/s40520-026-03417-0)
Supplement: Supplementary file 1 — Supplementary file1 [file 40520_2026_3417_MOESM1_ESM.docx]

**Association between angiogenic T cells and cognitive function in type 2 diabetes**

Miriam Longo^1,2^, Paola Caruso^3^, Maria Chiara Auriemma^2,4^, Antonietta Maio^3^, Irene Di Meo^2,4^, Lorenzo Scappaticcio^2^, Maria Ida Maiorino^2,3^, Giuseppe Bellastella^2,3^, Maria Rosaria Rizzo^2,4^, Giuseppe Paolisso^2,4^, Katherine Esposito^2,3^

^1^Department of Life Science, Health, and Health Professions, Link Campus University, Roma, Italy; ^2^Department of Advanced Medical and Surgical Sciences, University of Campania “Luigi Vanvitelli”, Naples, Italy;

^3^Division of Endocrinology and Metabolic Diseases, AOU University of Campania “Luigi Vanvitelli”, Naples, Italy;

^4^Division of Geriatrics and Internal Medicine, AOU University of Campania "Luigi Vanvitelli", Naples, Italy.

**Correspondence** to Miriam Longo, MD, ^1^Department of Life Science, Health, and Health Professions, Link Campus University, Roma, Italy; Department of Advanced Medical and Surgical Sciences, Division of Endocrinology and Metabolic Diseases, University of Campania “Luigi Vanvitelli”;

**Type of paper:** Research Article

**Supplementary file**

Supplementary Table S1 ………………………………………………………………………………………………pag. 2

**Supplementary table S1.** Main clinical characteristics in participants in the study according to the followed therapy (GLP-1RA + MET and MET groups), after propensity score matching.

|  | **GLP-1RA + MET group (N = 35)** | **MET group (N = 35)** | **P** |
| --- | --- | --- | --- |
| Age, years | 72 (67.5, 72) | 70 (68.2,75) | 0.915 |
| Duration of diabetes, years | 20 (11, 28) | 19 (12, 27) | 0.520 |
| Fasting glucose, mg/dl | 111 (110, 119) | 101 ( 93, 164) | 0.663 |
| HbA1c, % | 7.3 (7, 7.6) | 7.2 (6.8, 7.8) | 0.813 |
| Weight, Kg | 77.3 (69.5 89.6) | 76.5 (75, 90.2) | 0.444 |
| BMI, Kg/m^2^ | 29.1 (26.8–32.5) | 28.7 (27.5–33.0) | 0.356 |
| SBP, mmHg | 130 (120, 135) | 120 (120, 130) | 0.066 |
| DBP, mmHg | 80 (80, 80) | 80 (70,80) | 0.151 |
| eGFR, ml/min/1.73m^2^ | 89 (59, 100) | 82 (77, 90) | 0.916 |
| MoCA | 28 (28, 30) | 19 (17, 25) | <0.001 |
| MMSE  T ang (%) | 29 (29, 30)  22.5 (15.2, 27.6) | 25.9 (21.7, 22)  16.1 (12.7, 17.2) | <0.001  0.002 |

**Abbreviations:** BMI, body mass index; SBP, systolic blood pressure; DBP, diastolic blood pressure; eGFR, estimated glomerular filtration rate; MoCA, Montreal Cognitive Assessment; MMSE, Mini-Mental State Examination; HbA1c, glycated hemoglobin.
